# Long-term oncologic outcomes of radiotherapy combined with maximal androgen blockade for localized, high-risk prostate cancer

**DOI:** 10.1186/s12957-018-1395-5

**Published:** 2018-06-11

**Authors:** Yong Luo, Mingchuan Li, Hengzhi Qi, Jiahui Zhao, Yili Han, Yunhua Lin, Zhu Hou, Yongguang Jiang

**Affiliations:** Department of Urology, Beijing Anzhen Hospital, Capital Medical University, Anzhenli Street, Chaoyang District, Beijing, 100029 People’s Republic of China

**Keywords:** Prostate cancer, Brachytherapy, PSA kinetics, Maximal androgen blockade, Radiation therapy

## Abstract

**Background:**

To assess the oncologic outcomes of radiation therapy (RT) combined with maximal androgen blockade (MAB) and prostate-specific antigen (PSA) kinetics in patients with localized, high-risk prostate carcinoma (PCa).

**Methods:**

Three-hundred twenty individuals with localized PCa who underwent RT + MAB in 2001–2015 were evaluated retrospectively. All patients had received 36 months of MAB therapy and 45 Gy of pelvic irradiation, plus a dose-escalated external beam radiation therapy (DE-EBRT) boost to 76~81 Gy (MAB + EBRT group), or a low-dose-rate prostate permanent brachytherapy (LDR-PPB) boost to 110 Gy with I-125 (MAB + EBRT + PPB group).

**Results:**

Follow-up median is 90 months, ranging from 12 to 186 months; 117 (36.6%) and 203 (63.4%) cases underwent MAB + EBRT and MAB + EBRT + PPB, respectively. Multivariate Cox regression showed that the PPB regimen and PSA kinetics were positive indicators of oncologic outcomes. Compared with MAB + EBRT, MAB + EBRT + PPB remarkably improved PSA kinetics more pronouncedly: PSA nadir (1.3 ± 0.7 vs 0.11 ± 0.06 ng/mL); time of PSA decrease to nadir (7.5 ± 1.8 vs 3.2 ± 2.1 months); PSA doubling time (PSADT; 15.6 ± 4.2 vs 22.6 ± 6.1 months); decrease in PSA (84.6 ± 6.2% vs 95.8 ± 3.4%). Additionally, median times of several important oncologic events were prolonged in the MAB + EBRT + PPB group compared with the MAB + EBRT group: overall survival (OS; 12.3 vs 9.1 years, *P* < 0.001), biochemical recurrence-free survival (BRFS; 9.8 vs 6.5 years, *P* < 0.001), skeletal-related event (SRE; 10.4 vs 8.2 years, *P* < 0.001), and cytotoxic chemotherapy (CCT; 11.6 vs 8.8 years, *P* = 0.007).

**Conclusion:**

MAB + EBRT + PPB is extremely effective in patients with localized, high-risk PCa, indicating that PPB may play a synergistic role in improving PSA kinetics and independently predicts oncologic outcomes.

## Background

Prostate cancer (PCa) is a common malignancy characterized by both elevated morbidity and mortality. Most PCa patients, including those with high-risk disease, do not have metastatic tumors at diagnosis; therefore, local tumor resection could produce excellent long-term survival outcomes [1–3]. In individuals with localized, high-risk disease, not suitable for radical prostatectomy (RP), combination therapies involving RT and androgen deprivation therapy (ADT) are preferred approaches.

As a new RT technique, modern brachytherapy was first applied for PCa in the 1980s when transrectal ultrasound became available to plan and guide radioactive seed placement within the prostate. Because of excellent 15-year PSA outcomes [4], brachytherapy is routinely performed either as monotherapy in individuals with low-risk or low-/intermediate-risk cancer or in combination with external beam radiation therapy (EBRT) in those with high-risk tumors [5]. A recent comprehensive literature review screening 18,000 articles with over 50,000 patients comparatively analyzed PSA-free survival outcomes in patients suffering from localized PCa treated with different radical therapies [1]. The results suggested that PSA outcomes are significantly favorable after brachytherapy in comparison with EBRT in low-risk cases, with the brachytherapy monotherapy achieving equivalent PSA outcomes compared to the EBRT and brachytherapy combination in individuals with intermediate-risk tumors. Here, we evaluated the clinical benefit of the MAB + EBRT + PPB combination by assessing long-term survival outcomes and PSA kinetics in subjects with localized high risk.

## Methods

### Subjects

All patients with localized, high-risk PCa treated by RT plus 36 months of MAB therapy from 01/01/2001 to 06/30/2015 in our institution were enrolled in the present retrospective analysis. Some patients underwent the dose-escalated external beam radiation therapy (DE-EBRT) protocol of pelvic irradiation to 45 Gy and prostate irradiation to 76~81 Gy (MAB + EBRT group), while the remaining cases were administered combined RT protocol of pelvic irradiation to 45 Gy and LDR-PPB to 110 Gy (MAB + EBRT + PPB group).

The patients were clinically diagnosed by determining serum PSA levels, transrectal prostate ultrasonography, pathological examination of puncture biopsy specimens or surgically removed samples, radioisotope scan of the bone, and abdominal and pelvic computed tomography. Follow-up for all patients ended on 06/30/2016 in this retrospective clinical trial. Risk classification was based on the Memorial Sloan-Kettering group definition, in which patients are classified as having low (PSA ≤ 10 ng/mL, Gleason score ≤ 6, and clinical stage ≤ T2a), intermediate (PSA = 10–20 ng/mL, Gleason score = 7, and/or clinical stage T2b), and high (PSA ≥ 20 ng/mL, Gleason score ≥ 8, clinical stage ≥ T2c, and/or two to three intermediate-risk features) risk.

### Patient follow-up and data collection

Patients were monitored by serum PSA assessment quarterly for year 1, then at 6-month intervals for year 2, and once a year afterwards. During follow-up, we measured PSA kinetics, including PSA nadir, the time required for PSA to reach nadir, and PSA decrease. In addition, PSA doubling time (PSADT) was determined as previously reported [6]. Furthermore, radioisotope scan of the bone and computed tomography of the pelvis, lung, and skull were performed every year.

### Study endpoints

Primary study endpoints were OS (time elapsed from treatment to death) and BRFS (time to PSA biochemical recurrence). Secondary endpoints included SRE-free survival (SRE-FS; time to the first SRE) and CCT-free survival (CCT-FS; time to the first CCT). PSA kinetics was also assessed as described above. PSA biochemical recurrence was reflected by more than 1.25-fold elevation compared to baseline values (for cases with no previous PSA level decrease) or exceeding the nadir level (for the remaining cases), and absolute PSA amounts increased by ≥ 2 ng/mL [7]. Radiotherapy or bone surgery, pathologic bone fractures, spinal cord compression, and antineoplastic treatment changes for bone pain alleviation were generally considered SRE.

### Adverse effect assessment

In the form of telephone inquiring and questionnaire, we regularly monitored the complications of patients during the treatment process. Acute symptoms were related to radiation effects on proliferating tissues at the time of radiation treatment, and late symptoms occurred months after radiation treatment and were likely to remain. Additionally, we also observed the possible complications related to ADT on multiple system functions, such as endocrine symptoms, sexual function, cardiovascular events, and several important organ functions. Acute urogenital symptoms were classified according to the standard recommended by American Brachytherapy Society (ABS) as the following: grade 0, without any complication; grade 1, mild urination burning and frequency (2–3 times every night), no intervention required; grade 2, moderate urination burning and frequency (4–6 times every night) and gross hematuria, but conservative measures are generally effective; grade 3, severe urination burning and frequency (7–10 times every night) and gross hematuria, requiring active intervention; grade 4, severe hesitancy or retention, requiring catheterization. Acute rectal symptoms were evaluated using Radiation Therapy Oncology Group (RTOG) toxicity scoring criteria: grade 0, without any complication; grade 1, symptoms of rectal frequency, urgency, tenesmus or mucoid stool, which need to be treated with conservative measures; grade 2, intermittent rectal bleeding, rectum erythema, requiring active intervention; grade 3, rectal ulceration and severe bleeding, which would require emergent colonoscopy fulguration and blood transfusion; grade 4, intestinal obstruction or fistula, massive rectal bleeding, which need to be emergently treated with surgery or vascular support.

### Statistical analysis

Prognostic parameters were first evaluated by univariate (log-rank) and multivariate (Cox regression) analyses. Next, PSA kinetics was compared between the two treatment groups by independent sample *t* test. Furthermore, OS, BRFS, SRE-FS, and CCT-FS curves were obtained by the Kaplan-Meier method. To test the statistical significance of the difference in adverse effects between the two groups, chi-square test was done. *P* < 0.05 was considered to reflect statistical significance.

## Results

### Patients’ characteristics

In this study, 320 subjects with localized, high-risk PCa administered combination treatment of RT + MAB were included. Median follow-up was 90 months (12~186 months). Among the patients, 117 (36.6%) cases underwent MAB + EBRT and 203 (63.4%) received MAB + EBRT + PPB. The detailed clinical and treatment characteristics of the patients are provided in Table 1.

**Table 1:** Clinical and treatment characteristics of the patients

	Median	Range
Age at diagnosis (years)	70	58~81
Gland volume (mL)	33.4	27~62
Follow-up (months)	90	12~186
	Count	Percentage (%)
Clinical stage		
T2b	23	7.2
T2c	167	52.2
T3a	82	25.6
T3b	48	15
Gleason score		
≤6	3	0.9
7	11	3.4
≥8	306	95.6
PSA at diagnosis (ng/mL)		
≤10	35	10.9
10~20	49	15.3
≥20	236	73.8
Memorial Sloan-Kettering risk classification		
2~3 IS	71	22.2
1 HS	106	33.1
2~3 HS	143	44.7
MAB		
Continuous	184	57.5
Intermittent	136	42.5
PPB		
Yes	203	63.4
No	117	36.6
PSA nadir (ng/mL)		
≤1.0	241	75.3
>1.0	79	24.7
Time to PSA nadir (months)		
≤3	207	64.7
>3	113	35.3
PSA doubling time (months)		
≤12	46	14.4
>12	274	85.6
PSA decrease (%)		
<90	71	22.2
≥90	249	77.8

*MAB* Maximal Androgen Blockade; *PPB* Permanent Prostate Brachytherapy; *IS* intermediate-Risk Standard; *HS* High-Risk Standard

### Factors influencing survival prognosis

Table 2 summarizes univariate and multivariate analyses for OS predictors. Univariate analysis indicated that age (*P* = 0.035, hazard ratio [HR] 5.812), gland volume (*P* = 0.006, HR 3.816), PPB addition (*P* = 0.007, HR 3.016), clinical stage (*P* < 0.001, HR 4.557), Gleason score (*P* = 0.001, HR 3.356), baseline PSA (*P* = 0.027, HR 1.558), Memorial Sloan-Kettering Risk Classification Standard score (*P* < 0.001, HR 7.658), PSA nadir (*P* < 0.001, HR 9.473), time to PSA nadir (*P* = 0.012, HR 3.113), PSADT (*P* = 0.042, HR 2.665), and PSA level reduction (*P* < 0.001, HR 13.463) were significant predictors of OS in patients with localized, high-risk PCa. Multivariate Cox regression analysis further identified gland volume (*P* = 0.042, HR 1.192), PPB addition (*P* < 0.001, HR 6.358), clinical stage (*P* = 0.011, HR 2.183), Gleason score (*P* < 0.001, HR 7.142), baseline PSA (*P* = 0.014, HR 3.492), Memorial Sloan-Kettering Risk Classification Standard score (*P* < 0.001, HR 5.479), PSA nadir (*P* = 0.012, HR 4.553), time to PSA nadir (*P* = 0.038, HR 1.249), PSADT (*P* = 0.028, HR 5.511), and PSA reduction (*P* < 0.001, HR 7.845) as independent prognostic indicators of OS

**Table 2:** Analyses for prognostic indicators of Overall Survival

Variable	Overall Survival			
	Univariate analysis		Multivariate analysis	
	P Value	HR	P Value	HR
Age at diagnosis (years) ≤70 vs >70	0.035	5.812	0.174	――
Gland volume (mL) ≤33 vs >33	0.006	3.816	0.042	1.192
MAB Continuous vs Intermittent	0.583	――	0.457	――
PPB yes vs no	0.007	3.016	<0.001	6.358
Clinical stage ≤ T2c vs ≥T3a	<0.001	4.557	0.011	2.183
Gleason score ≤7 vs ≥8	0.001	3.356	<0.001	7.142
PSA at diagnosis (ng/mL) ≤10 vs 10~20 vs ≥20	0.027	1.558	0.014	3.492
Memorial Sloan-Kettering risk classification 2~3 IS vs 1 HS vs 2 HS	<0.001	7.658	<0.001	5.479
PSA nadir (ng/mL) ≤1 vs >1	<0.001	9.473	0.012	4.553
Time to PSA nadir (months) ≤3 vs >3	0.012	3.113	0.038	1.249
PSA doubling time (months) ≤12 vs >12	0.042	2.665	0.028	5.511
PSA decrease (%) <90 vs ≥90	<0.001	13.463	<0.001	7.845

*MAB* Maximal Androgen Blockade; *PPB* Permanent Prostate Brachytherapy; *IS* intermediate-Risk Standard; *HS* High-Risk Standard

The prognostic indicators of BRFS are presented in Table 3. Univariate analysis indicated that gland volume (*P* = 0.047, HR 3.668), MAB pattern (*P* = 0.031, HR 1.492), PPB addition (*P* = 0.001, HR 2.888), clinical stage (*P* = 0.041, HR 4.737), Gleason score (*P* = 0.013, HR 5.711), baseline PSA (*P* = 0.019, HR 2.622), Memorial Sloan-Kettering Risk Classification Standard score (*P* = 0.027, HR 1.772), PSA nadir (*P* = 0.041, HR 1.323), time to PSA nadir (*P* = 0.032, HR 2.116), PSADT (*P* = 0.048, HR 1.863), and PSA reduction (*P* = 0.006, HR 3.677) were significant predictors of BRFS in patients with localized, high-risk PCa. Multivariate Cox regression analysis further identified gland volume (*P* = 0.016, HR 8.336), MAB pattern (*P* = 0.018, HR 3.217), PPB addition (*P* < 0.001, HR 5.126), clinical stage (*P* = 0.013, HR 6.142), Gleason score (*P* = 0.022, HR 3.463), PSA baseline (*P* = 0.001, HR 6.334), Memorial Sloan-Kettering Risk Classification Standard score (*P* = 0.009, HR 3.643), PSA nadir (*P* = 0.016, HR 6.993), time to PSA nadir (*P* = 0.011, HR 5.843), PSADT (*P* = 0.014, HR 6.132), and PSA reduction (*P* < 0.001, HR 9.385) as independent prognostic indicators of BRFS.

**Table 3:** Analyses for prognostic indicators of Biochemical Recurrence-Free Survival

Variable	Biochemical Recurrence-Free Survival			
	Univariate analysis		Multivariate analysis	
	P Value	HR	P Value	HR
Age at diagnosis (years) ≤70 vs >70	0.339	――	0.147	――
Gland volume (mL) ≤33 vs >33	0.047	3.668	0.016	8.336
MAB Continuous vs Intermittent	0.031	1.492	0.018	3.217
PPB yes vs no	0.001	2.888	<0.001	5.126
Clinical stage ≤ T2c vs ≥T3a	0.041	4.737	0.013	6.142
Gleason score ≤7 vs ≥8	0.013	5.711	0.022	3.463
PSA at diagnosis (ng/mL) ≤10 vs 10~20 vs ≥20	0.019	2.622	0.001	6.334
Memorial Sloan-Kettering risk classification 2~3 IS vs 1 HS vs 2 HS	0.027	1.772	0.009	3.643
PSA nadir (ng/mL) ≤1 vs >1	0.041	1.323	0.016	6.993
Time to PSA nadir (months) ≤3 vs >3	0.032	2.116	0.011	5.843
PSA doubling time (months) ≤12 vs >12	0.048	1.863	0.014	6.132
PSA decrease (%) <90 vs ≥90	0.006	3.677	<0.001	9.385

*MAB* Maximal Androgen Blockade; *PPB* Permanent Prostate Brachytherapy; *IS* intermediate-Risk Standard; *HS* High-Risk Standard

### Characteristics of high-risk patients treated with different RT regimens

To assess how different RT regimens affect the PSA kinetics and oncologic outcomes, we further divided the high-risk patients into two different treatment groups, whose clinical and pathological characteristics are summarized in Table 4.

**Table 4:** Comparison of the characteristics of high-risk patients undergone different treatment

Treatment	MAB+EBRT (n=117)	MAB+EBRT+PPB (n=203)	
	Median (Range)	Median (Range)	P Value
Age at diagnosis (years)	70 (59~81)	69 (58~79)	0.11
Gland volume (mL)	32.3 (27~62)	34.6 (29~62)	0.09
Follow-up (months)	84 (12~186)	90 (12~186)	0.25
	Count (%)	Count (%)	P Value
Clinical stage			
T2b	9 (7.7)	14 (6.9)	0.32
T2c	63 (53.8)	104 (51.2)	
T3a	28 (23.9)	54 (26.6)	
T3b	17 (14.5)	31 (15.3)	
Gleason score			
≤6	2 (1.7)	1 (0.5)	0.27
7	3 (2.6)	8 (3.9)	
≥8	112 (95.7)	194 (95.6)	
PSA at diagnosis (ng/mL)			
≤10	17 (14.5)	18 (8.9)	0.13
10~20	19 (16.2)	30 (14.8)	
≥20	81 (69.2)	155 (76.4)	
Memorial Sloan-Kettering risk classification			
2~3 IS	27 (23.1)	44 (21.7)	0.61
1 HS	38 (32.5)	68 (33.5)	
2~3 HS	52 (44.4)	91 (44.8)	
MAB			
Continuous	58 (49.6)	126 (62.1)	0.07
Intermittent	59 (50.4)	77 (37.9)	

*MAB* Maximal Androgen Blockade; *PPB* Permanent Prostate Brachytherapy; *IS* Intermediate-Risk Standard; *HS* High-Risk Standard

### PSA kinetics in high-risk patients treated with different RT regimens

**Fig. 1:**
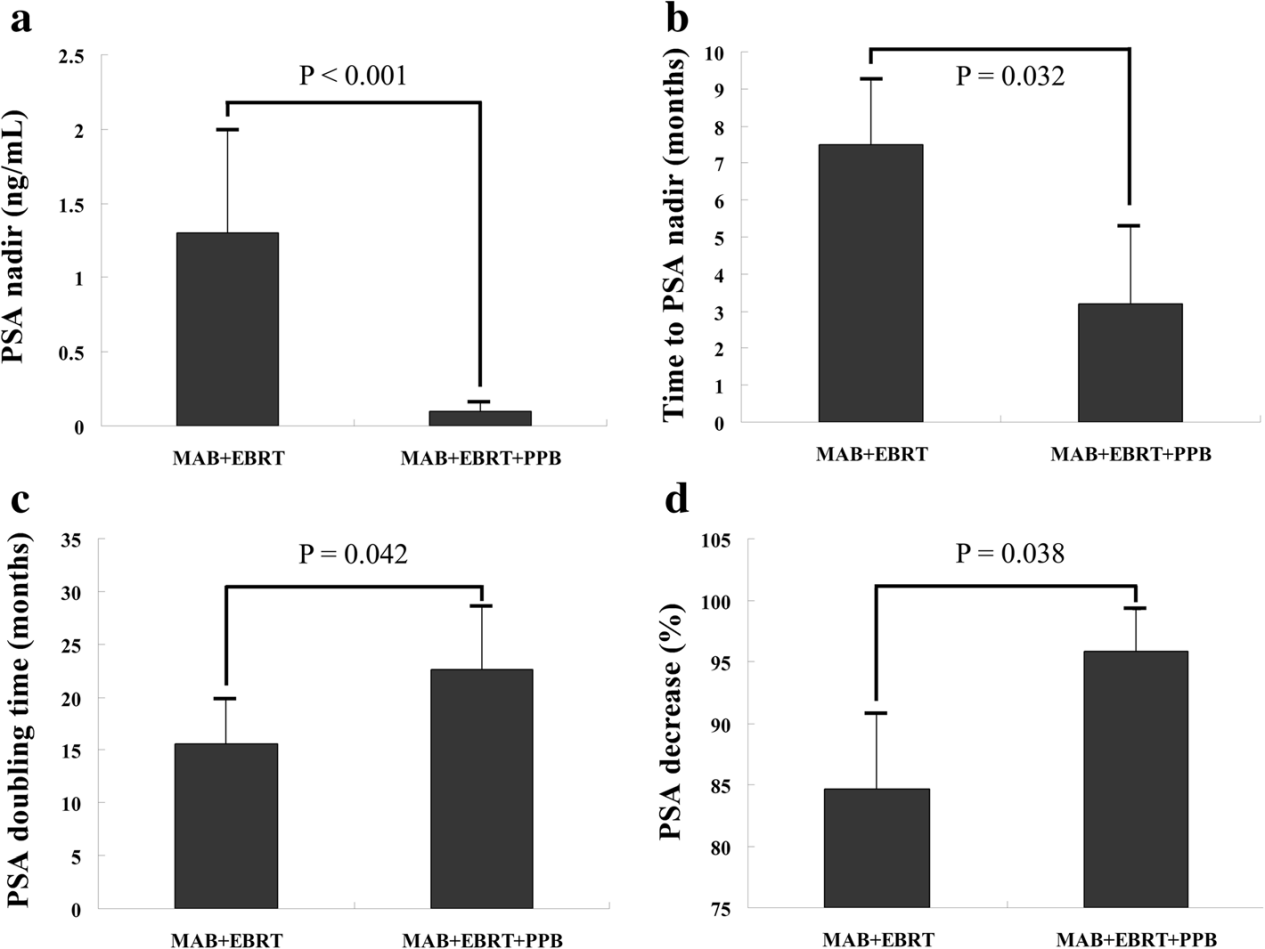
Differences in PSA kinetics between cases treated by MAB + EBRT and MAB + EBRT + PPB combination therapies. **a** PSA nadir. **b** Time to PSA nadir. **c** PSA doubling time. **d** Declining extent of PSA

As shown in Fig. 1, the parameters of PSA kinetics were affected by different RT regimens in patients with localized, high-risk PCa. PSA nadir values in the MAB + EBRT and MAB + EBRT + PPB combination groups were 1.3 ± 0.7 ng/mL (range, 0.03–14.5 ng/mL) and 0.11 ± 0.06 ng/mL (range, 0.00–1.27 ng/mL), respectively. Times of PSA decrease to nadir in these two groups were 7.5 ± 1.8 months (range, 3–12 months) and 3.2 ± 2.1 months (range, 1–9 months), respectively. Meanwhile, PSADT values were 15.6 ± 4.2 months (range, 7.1–27.4 months) in the MAB + EBRT group and 22.6 ± 6.1 months (range, 7.6–43.2 months) in the MAB + EBRT + PPB group. Finally, PSA levels were reduced by 84.6 ± 6.2% (range, 67.1–94.5%) in the MAB + EBRT group and 95.8 ± 3.4% (range, 83.1–99.99%) in the MAB + EBRT + PPB group. These findings demonstrated that PSA kinetics, which is an important independent indicator of OS, and BRFS could be notably improved by PPB-based combination regimens.

### Endpoint events in high-risk patients treated with different RT regimens

**Fig. 2:**
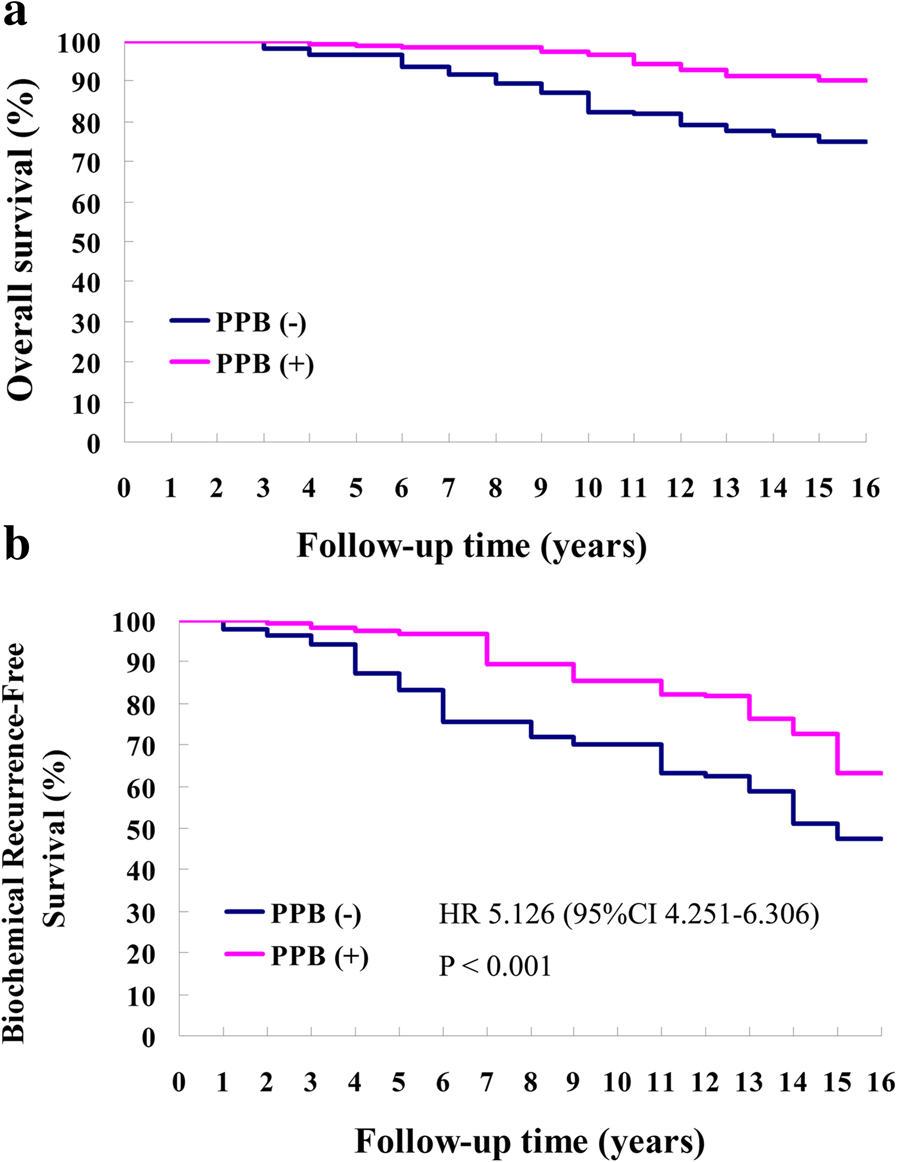
**a** Overall survival of cases administered MAB + EBRT and MAB + EBRT + PPB combination therapies. **b** PSA biochemical recurrence-free survival of cases administered MAB + EBRT and MAB + EBRT + PPB combination therapies

As shown in Fig. 2a, the 5-, 7-, 10-, 12-, and 15-year overall survival rates in the MAB + EBRT + PPB group were markedly higher than those in the MAB + EBRT group (99.4 vs 96.6%, *P* = 0.241; 98.3 vs 93.4%, *P* = 0.039; 97.2 vs 87.3%, *P* = 0.011; 94.5 vs 81.8%, *P* = 0.003; 91.4 vs 76.5%, *P* < 0.001). Median OS was 9.1 years [95% confidence interval (CI) 7.5 to 12.6] among patients receiving MAB + EBRT and 12.3 years (95% CI 10.6 to 13.2) in those administered MAB + EBRT + PPB (HR 6.358, 95% CI 5.733 to 6.627, *P* < 0.001). Meanwhile, the PPB-based combination regimen significantly increased the median time of PSA biochemical progression, from 6.5 years (95% CI 4.8 to 8.1) in the MAB + EBRT group to 9.8 years (95% CI 8.5 to 10.7) in MAB + EBRT + PPB-treated patients (HR 5.126, 95% CI 4.251 to 6.306, *P* < 0.001) (Fig. 2b).

**Fig. 3:**
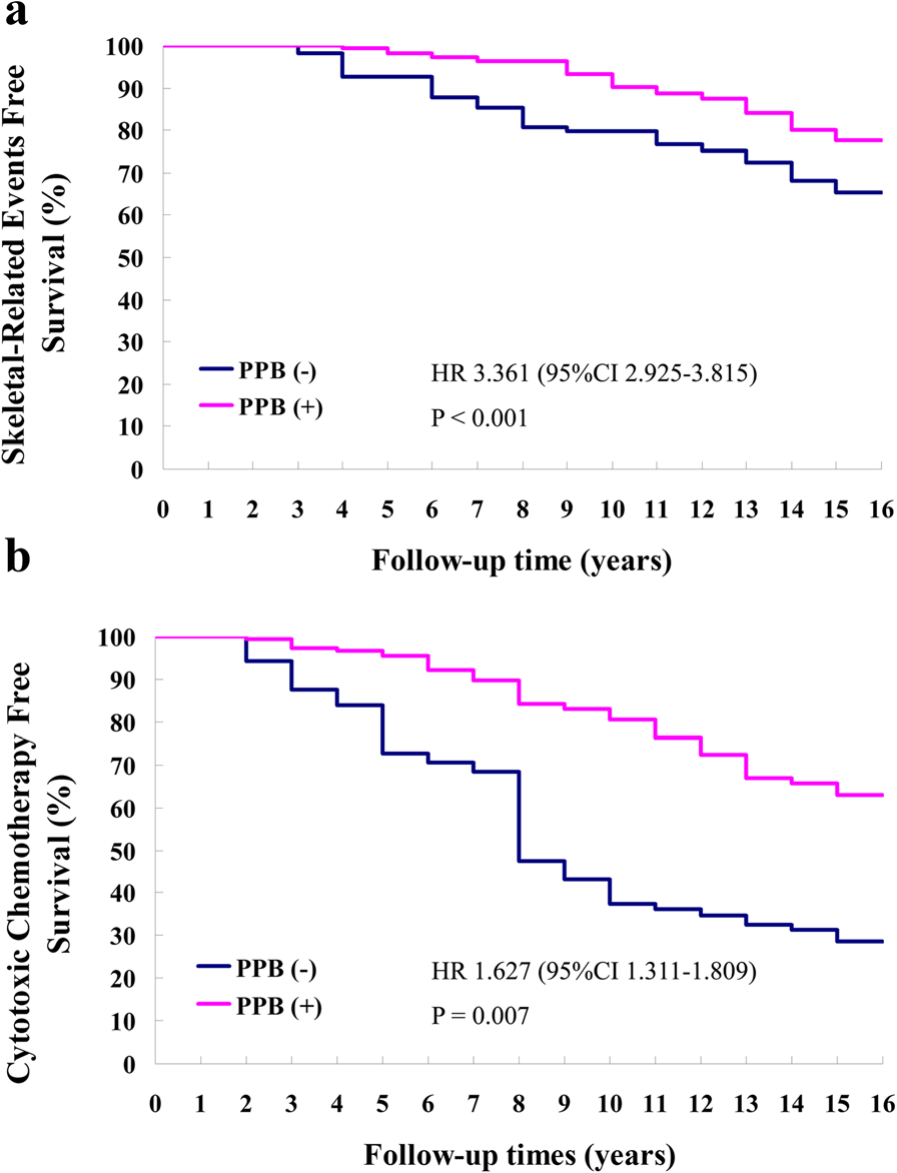
**a** Skeletal-related event-free survival of cases administered MAB + EBRT and MAB + EBRT + PPB combination therapies. **b** Cytotoxic chemotherapy-free survival of cases administered MAB + EBRT and MAB + EBRT + PPB combination therapies

A median time to first SRE of 10.4 years (95% CI 8.9 to 12.2) was found in the MAB + EBRT + PPB group, compared with 8.2 years (95% CI 7.1 to 10.5) in MAB + EBRT-treated individuals, indicating significantly reduced risk of SRE (HR 3.361, 95% CI 2.925 to 3.815, *P* < 0.001) (Fig. 3a). The superiority of MAB + EBRT + PPB over MAB + EBRT was also shown for CCT initiation (Fig. 3b). Indeed, median times to CCT initiation were 11.6 years (95% CI 9.8 to 12.7) and 8.8 years (95% CI 6.3 to 10.9 in the MAB + EBRT + PPB and MAB + EBRT groups, respectively. Treatment with the PPB-based combination regimen could remarkably increase the CCT-FS rate compared with MAB + EBRT (HR 1.627, 95% CI 1.311 to 1.809, *P* = 0.007).

### Complications in high-risk patients treated with different RT regimens

As shown in Table 5, the complication rates between MAB + EBRT group and MAB + EBRT + PPB group showed no significant differences in late radiation-related symptoms and multiple organ functions.

**Table 5:** Complications of high-risk patients undergone different treatment

Adverse Effects	MAB+EBRT %(n=117)	MAB+EBRT+PPB %(n=203)	χ ²	P Value
Acute Urology Function				
ABS Grade 0	12.82	5.42	5.447	0.020
ABS Grade 1	48.72	25.12	18.480	0.000
ABS Grade 2	20.51	31.53	4.516	0.034
ABS Grade 3	5.13	23.15	17.449	0.000
ABS Grade 4	12.82	14.78	0.235	0.628
Late Urology Function				
Urgent/Incontinence	1.71	1.48	0.026	0.872
Hesitancy/Retention	1.71	3.45	0.821	0.365
Gross Hematuria	4.27	6.4	0.635	0.426
Stricture	0	0.49	0.578	0.447
Frequency/Nocturia	15.38	25.12	4.162	0.041
Acute Gastrointestinal Function				
RTOG Grade 0	90.6	88.67	0.291	0.590
RTOG Grade 1	5.13	5.91	0.086	0.769
RTOG Grade 2	3.42	3.45	0.000	1.000
RTOG Grade 3	0.85	1.97	0.601	0.438
RTOG Grade 4	0	0	——	——
Late Gastrointestinal Function				
Diarrhoea	21.37	13.3	3.549	0.060
Nausea/Vomiting	7.69	4.93	1.017	0.313
Abdominal Pain	4.27	3.94	0.021	0.885
Rectal Bleeding	5.13	10.34	2.614	0.106
Intestinal Fistula	0	0.49	0.578	0.447
Endocriology Function				
Breast Pain	69.23	63.05	1.250	0.264
Xerosis Cutis	11.11	19.7	3.966	0.046
Hot Flush	20.51	37.44	9.897	0.002
Sexual Dysfunction	53.85	47.78	1.091	0.296
Other System Function				
Liver Dysfunction	5.13	8.37	1.172	0.279
Renal Dysfunction	7.69	6.4	0.192	0.661
Angina Pectoris	34.18	25.12	2.997	0.083
Heart Failure	7.69	2.96	3.727	0.054
Dyspnea	3.42	0.99	2.389	0.122
Anaemia(moderate/severe)	16.24	16.75	0.014	0.906

Note: *ABS* American Brachytherapy Society, *RTOG* Radiation Therapy Oncology Group

Although the group of MAB + EBRT + PPB patients displayed significant higher complications rates than those of MAB + EBRT cases in grade 2 (31.53 vs 20.51%, *P* = 0.034) and grade 3 (23.15 vs 5.13%, *P* < 0.001) of acute urogenital symptoms, all these symptoms could be improved gradually. Fifteen patients (12.82%) in MAB + EBRT group and 30 patients (14.78%) in MAB + EBRT + PPB group were identified as ABS grade 4 because of retention and catheterization, and catheter could be removed in the vast majority of these cases successfully. One patient (0.85%) in MAB + EBRT group and four patients (1.97%) in MAB + EBRT + PPB group were diagnosed as RTOG grade 3 due to rectal ulceration and severe bleeding, which were successfully treated with colonoscopy fulguration. And none of all patients developed to symptoms of RTOG grade 4. Additionally, only one case in the group of MAB + EBRT + PPB developed to intestinal fistula and received repair surgery.

In all, the combination therapy of MAB + EBRT + PPB showed similar safety to MAB + EBRT regimen, and no significant serious complications were observed in MAB + EBRT + PPB regimen.

## Discussion

In the past 20 years, interstitial radiation therapy has been used as routine treatment for patients with clinically localized PCa, and most researchers believe that the 5-year PSA outcome of brachytherapy in low-risk patients is not statistically different from that of RP or EBRT. In addition, intermediate- and high-risk patients administered RP or EBRT may show a better response compared with those that undergo brachytherapy [8]. However, this view remains controversial. Polascik et al. reported that 7-year actuarial PSA progression-free survival following RP is remarkably higher than that of the I-125 brachytherapy group (97.8 vs 79%) [9, 10] in patients with localized PCa. Therefore, Polascik et al. proposed that brachytherapy should be cautiously recommended to patients with localized PCa. Sharkey and colleagues analyzed 1707 PCa patients with T1 or T2 stage disease treated by either brachytherapy or RP; they concluded that the time to PSA-indicated recurrence is better controlled by brachytherapy than RP in intermediate- (89 vs 58%, *P* < 0.05) and high-risk (88 vs 43%, *P* < 0.05) groups, but not in low-risk patients (89 vs 94%, *P* = 0.174) [11]. Moreover, Taira et al. evaluated 329 cases of high-risk PCa treated with brachytherapy + EBRT with a 10-year follow-up and found that cause-specific survival (CSS) in Gleason 5 patients is significantly lower than that of non-Gleason 5 patients (90.3 vs 98.1%, *P* = 0.011). However, no remarkable differences in BRFS and OS between these two groups of patients were observed [12]. In addition, Demanes et al. retrospectively assessed 209 cases treated with brachytherapy **+** EBRT with a 10-year follow-up and reported OS and CSS rates of 79 and 97%, respectively. Meanwhile, PSA progression-free survival rates were different for patients with low-, intermediate-, and high-risk disease (90, 87, and 69%, respectively) [13]. Another study reported that the combination strategy of brachytherapy + EBRT is significantly more advantageous than brachytherapy monotherapy in 5-year biochemical relapse-free survival (80 vs 59%, *P* < 0.01), although EBRT-treated cases showed more adverse disease factors [14]. Collectively, current clinical evidence supports brachytherapy + EBRT as a proven treatment regimen for all stages of localized PCa [15].

In the present analysis, EBRT + MAB + PPB showed a significant benefit for long-term OS and BRFS in localized high-risk patients compared to EBRT + MAB combination. In addition, the brachytherapy-based combination treatment also postponed several important clinical events: 3.3 years for PSA biochemical recurrence, 2.2 years for SRE, and 2.8 years for CCT. The recently open-published ASCENDE-RT trial [16] compared survival endpoints between the DE-EBRT and low-dose-rate brachytherapy (LDR-BT) arms in intermediate-/high-risk patients and showed that DE-EBRT is twice as likely to result in biochemical failure, while OS rates were similar between these two treatment arms (*P* = 0.62).

Recent studies reported that PSA kinetics is closely related to long-term survival outcomes in PCa patients [17, 18]. More importantly, PSA kinetics was confirmed to independently predict OS and BRFS by multivariate analysis in the current analysis. Specifically, PSA reduction over 90% was strongly associated with improved long-term survival as well as PSA biochemical progression in high-risk disease cases treated with RT + MAB. It is known that a short PSADT is associated with a promptly expanding tumor, a higher metastatic potential, and a somewhat elevated risk of cancer specific mortality [19–21]. D’Amico et al. found that patients with a PSADT less than 3 months represent 10–15% of males showing biochemical recurrence, but a higher risk of systemic recurrence [20, 21] and cancer-specific mortality with a median survival of 6 years [22]. Similarly, in men post-RT, Crook et al. demonstrated systemic recurrence is correlated with elevated PSA nadir, as well as reduced PSADT; an average PSA nadir of 0.4 ng/mL in cases without disease recurrence was reached at 33 months, while 3.2, 7.7, and 1.4 ng/mL were obtained in individuals with local recurrence at 17 months, distant recurrence at 12 months, and biochemical recurrence at 24 months, respectively [23].

In recent years, high-dose-rate brachytherapy (HDR-BT) has attracted increasing attention and is used for more patients. Several clinical trials reported its excellent effects in high-risk patients. Ten-year actuarial biochemical control rates of 100, 91, 88, and 79% were found in subjects with low-, two intermediate-, only one high-, and 2–3 high-risk criteria, respectively (*P* = 0.004); hormone treatment did not affect these results [24]. A 5-year BRFS of 93.6% was reported in high-risk patients who underwent ADT + EBRT + HDR-BT, with 87.6% in the EBRT + HDR-BT group [25]. The overall 3-year OS and BRFS rates were 93.7 and 96.9% in high-risk cases administered ADT + EBRT + HDR-BT, respectively [26]. Satoshi et al. reported that both LDR-BT + EBRT and HDR-BT + EBRT are safe and suitable for individuals with localized prostate carcinoma, with some advantages of HDR-BT + EBRT over LDR-BT + EBRT in terms of recovery time [27]. Although no further long-term survival data were reported for these two radiation modalities in localized, high-risk patients, LDR-BT is generally administered as a monotherapy in early diagnosed cases, while HDR-BT is usually applied along with EBRT in cases of prostate cancer in unspecified stages [28]. In addition, some clinical trials found that a PSA nadir of less than 0.02 ng/mL within 12 months of radiotherapy is associated with significantly improved biochemical tumor control and cause-specific survival in cases of locally advanced and non-metastatic high-risk prostate cancer co-administered HDR-BT, EBRT, and long-term ADT [29]. Thorsten et al. reported a discrepant conclusion regarding the predictive value of PSA for the biochemical control rate in 79 cases with high-risk PCa administered HDR-BT following EBRT, with an average follow-up of 21 months; the authors described PSA as a negative predictive biomarker for local recurrence during follow-up, indicating that prolonged follow-up is required for reassessing long-term outcomes [30].

## Conclusion

Overall, brachytherapy is a promising and effective radiation technique, with higher concentration of the radiation dose within the prostate, which decreases the risk of complications in other organs and reduces the frequency of urinary symptoms. PPB-based combined radiotherapy plays an extremely important role in improving OS and BRFS in high-risk PCa patients; time to the first SRE and CCT were also relatively prolonged. These clinical data further demonstrate that post-radiation PSA kinetics could significantly predict survival outcomes in cases with localized, high-risk disease; specifically, PSA nadir ≤ 1 ng/mL, time to PSA nadir ≤ 3 months, PSA doubling time > 12 months, and PSA reduction ≥ 90% were associated with improved tumor control. Therefore, more aggressive treatments should be considered for cases with non-favorable PSA kinetics.

## Abbreviations

ADT: Androgen deprivation therapy
BRFS: Biochemical recurrence-free survival
CCT: Cytotoxic chemotherapy
CCT-FS: Cytotoxic chemotherapy-free survival
CI: Confidence interval
CSS: Cause-specific survival
DE-EBRT: Dose-escalated external beam radiation therapy
HDR-BT: High-dose-rate brachytherapy
LDR-BT: Low-dose-rate brachytherapy
LDR-PPB: Low-dose-rate permanent prostate brachytherapy
MAB: Maximal androgen blockade
OS: Overall survival
PCa: Prostate cancer
PSA: Prostate-specific antigen
PSADT: PSA doubling time
RP: Radical prostatectomy
RT: Radiation therapy
SRE: Skeletal-related event
SRE-FS: Skeletal-related event-free survival
